# Genome-Wide Insights into Internalizing Symptoms in Admixed Latin American Children

**DOI:** 10.3390/genes16010063

**Published:** 2025-01-08

**Authors:** Gabriela de Sales Guerreiro Britto, Alberto O. Moreira, Edson Henrique Bispo Amaral, Daniel Evangelista Santos, Raquel B. São Pedro, Thaís M. M. Barreto, Caroline Alves Feitosa, Darci Neves dos Santos, Eduardo Tarazona-Santos, Maurício Lima Barreto, Camila Alexandrina Viana de Figueiredo, Ryan dos Santos Costa, Ana Lúcia Brunialti Godard, Pablo Rafael Silveira Oliveira

**Affiliations:** 1 Instituto de Biologia, Universidade Federal da Bahia, Salvador 40170-115, Brazil; gabritoo.bio@gmail.com (G.d.S.G.B.); deltaalberto@hotmail.com (A.O.M.); riky.bispo.amaral@gmail.com (E.H.B.A.); danielevansantos@gmail.com (D.E.S.); bispo.raquel1@gmail.com (R.B.S.P.); thaismaiamb@gmail.com (T.M.M.B.); 2 Escola Bahiana de Medicina e Saúde Pública, Salvador 40295-150, Brazil; feitosacaroline@gmail.com; 3 Instituto de Saúde Coletiva, Universidade Federal da Bahia, Salvador 40110-040, Brazil; darci@ufba.br; 4 Departamento de Genética, Ecologia e Evolução, Instituto de Ciências Biológicas, Universidade Federal de Minas Gerais, Belo Horizonte 31270-901, Brazil; edutars@gmail.com (E.T.-S.); brunialt@ufmg.br (A.L.B.G.); 5 Centro de Integração de Dados e Conhecimentos para Saúde, Fundação Oswaldo Cruz, Salvador 41745-715, Brazil; mauricio.barreto@fiocruz.br; 6 Instituto de Ciências da Saúde, Universidade Federal da Bahia, Salvador 40231-300, Brazil; cavfigueiredo@gmail.com (C.A.V.d.F.); ryan.costa@ufba.br (R.d.S.C.)

**Keywords:** internalizing symptoms, GWAS, childhood, Latin Americans

## Abstract

Background/Objectives: Internalizing disorders, including depression and anxiety, are major contributors to the global burden of disease. While the genetic architecture of these disorders in adults has been extensively studied, their early-life genetic mechanisms remain underexplored, especially in non-European populations. This study investigated the genetic mechanisms underlying internalizing symptoms in a cohort of Latin American children. Methods: This study included 1244 Brazilian children whose legal guardians completed the Child Behavior Checklist (CBCL) questionnaire. Genotyping was performed using the Illumina HumanOmni 2.5-8v1 BeadChip. Results: The genome-wide association analysis revealed a significant association of rs7196970 (*p* = 4.5 × 10^−8^, OR = 0.61), in the *ABCC1* gene, with internalizing symptoms. Functional annotation highlighted variants in epigenetically active regulatory regions, with multiple variants linked to differential expression of *ABCC1* across several human tissues. Pathway enrichment analysis identified 42 significant pathways, with notable involvement in neurobiological processes such as glutamatergic, GABAergic, and dopaminergic synapses. Conclusions: This study identifies *ABCC1* variants as novel genetic factors potentially associated with early-life internalizing symptoms. These results may contribute to future research on targeted interventions for childhood internalizing conditions.

## 1. Introduction

Mental disorders are characterized by significant disturbances in cognition, emotional regulation, or behavior which indicate dysfunction in psychological, biological, or developmental processes [1]. These conditions often result in profound distress and impair daily life activities. Among these, internalizing disorders refer to a broad class of conditions primarily characterized by negative emotional states that are experienced internally [2]. These phenotypes encompass a spectrum of emotional dysregulations, with depression and anxiety being the most prevalent and well-studied manifestations within this group [3]. Notably, depression and anxiety represent the leading causes of disability and the combined burden of disability and mortality worldwide [2,3]. In this context, Brazil has the highest prevalence of depression in Latin America and ranks second across the Americas in terms of overall depression rates [3].

Despite the global increase in the prevalence of mental disorders among children and adolescents, with rates reaching 20% in North America, 12% in Europe and Asia, and 8% in Africa [4,5], significant regional disparities persist in research, diagnosis, and treatment [6,7]. Early identification and treatment of internalizing symptoms in childhood are crucial for improving emotional regulation and preventing the progression to more severe disorders in the short term [8]. Moreover, addressing these symptoms early significantly reduces the long-term risk of developing chronic mental health issues, such as depression and anxiety [9].

Notably, a significant proportion of internalizing disorders diagnosed in adults have their onset in childhood [10]. During this critical developmental period, several environmental factors—including a history of emotional abuse [11], domestic and community violence [12], low socioeconomic status [13], and maternal behavior [14]—are linked to the development of internalizing symptoms. Other variables also contribute to the development of these phenotypes, with genetics accounting for substantial proportions of the heritability attributed to depression and anxiety in childhood (around 60–70%) [15,16]. In this context, the influence of environmental factors appears to increase with age, but more transiently. In contrast, genetic factors play a pivotal role in the long-term stability of these conditions [16].

Evidence suggests that a family history of depression and/or anxiety is associated with increased susceptibility and more severe disease profiles [17]. In this context, several genetic variants in genes encoding components of neurotransmission pathways have been associated with depression [18]. Furthermore, meta-analyses of genome-wide association studies (GWAS) identified dozens of loci significantly associated with depression [19,20]. These loci harbored genes encoding products involved in multiple biological functions, including neurotransmission, neurogenesis, and immune response. Recently, multi-ancestry GWAS have expanded our understanding of the genetic risk and pathogenesis of depression [21] and anxiety [22] disorders, particularly in populations of non-European ancestry.

Although many genomic studies have focused on internalizing disorders in adults, research on the genetic contributions to early-life symptoms remains limited, especially in non-Europeans. Current GWAS efforts have only focused on European ancestry cohorts, leaving a critical gap in understanding the genetic architecture of internalizing disorders in diverse populations. In this context, findings from a meta-analysis suggest that internalizing symptoms in children are influenced by multiple small-effect genetic variants, which overlap with those associated with other psychiatric disorders in both childhood and adulthood [23]. More recently, a study on the genetic architecture of internalizing symptoms in children and adolescents revealed significant genetic correlations with adult internalizing disorders and other childhood psychiatric traits, providing insights into the persistence of these symptoms over time [24]. Expanding these efforts to underrepresented populations is essential to ensure that the genetic findings are generalizable and to uncover ancestry-specific risk variants.

Given the limited understanding of the genetic basis of internalizing disorders in childhood, especially among non-European populations such as Latinos and African Americans, we conducted a genome-wide approach to explore the genetic architecture of this condition in 1244 admixed children from Brazil. Identifying the genetic factors involved in childhood internalizing symptoms could provide valuable insights into the progression of these disorders into adulthood.

## 2. Methods

### 2.1. Recruitment of Study Participants and Ethical Considerations

The study was conducted with children recruited as part of the SCAALA (Social Changes, Asthma, and Allergy in Latin America) project in Salvador, Brazil, comprising 1445 individuals aged 4 to 11 years [25]. The SCAALA study was approved by the research ethics committee of the Institute of Public Health at the Federal University of Bahia (003-05/CEP-ISC; Approval date: February 2005). The present study is part of the EPIGEN-Brazil project, whose study protocol was approved by the National Research Ethics Commission (CONEP, resolution: 15895/2011; Approval date: April 2013). Informed consent was obtained from the legal guardians of all participants for their involvement in interviews, blood collection, and genotyping procedures. All methods and protocols were carried out under the principles of the Declaration of Helsinki.

### 2.2. Psychological Assessment Tool

The Child Behavior Checklist (CBCL) [26] was applied to assess internalizing symptoms within the SCAALA cohort. The appropriate version of the CBCL was used based on the individual’s age: the Preschool Checklist (CBCL 1.5–5 years) or the School-age Checklist (CBCL 6–18 years). This tool consists of a 118-item questionnaire, with responses rated as 0 (never), 1 (sometimes), and 2 (always). The scores for each item related to internalizing symptoms were summed to produce a total raw score, which was then converted into a T score to reflect the intensity of the individual’s symptoms. The CBCL, translated and validated into Brazilian Portuguese by Bordin and colleagues [27], demonstrates high sensitivity, identifying 95% of moderate cases and 100% of severe childhood behavioral disorders.

Children with a CBCL T-score ≥ 64 were classified as exhibiting internalizing symptoms (INT), while those with a T-score < 64 were categorized as not exhibiting internalizing symptoms (N-INT). This threshold was based on recommendations from a previous study [27].

### 2.3. Genotyping and Quality Control

As part of the EPIGEN-Brazil consortium, individuals from the SCAALA cohort were randomly selected and genotyped for approximately 2.3 million Single Nucleotide Variants (SNVs) using the Illumina HumanOmni 2.5-8v1 BeadChip platform (Illumina, San Diego, CA, USA). In the present study, quality control (QC) procedures were performed to exclude SNVs and low-quality samples using the PLINK software (version 1.9). Variants or samples with a genotyping rate below 99% or SNVs that showed a significant deviation from Hardy–Weinberg equilibrium (*p* < 10^−5^) were excluded from further analyses. Furthermore, variants with a Minor Allele Frequency (MAF) < 1% were excluded from the study. After the QC, 1244 individuals (450 INT and 794 N-INT) and 1,758,937 genotyped autosomal variants remained in the study.

### 2.4. Genotype Imputation

Genotype imputation was performed as described by Magalhães and colleagues [28], using the EPIGEN-5M+1KGP reference panel. This panel integrates the 1000 Genomes Project haplotypes (phase 3, version 20130502), and our EPIGEN-5M panel, comprising 4,102,271 SNVs for 265 Brazilians. Strand alignment between the target dataset and the reference panel was verified with SHAPEIT2 [29], and strand inconsistencies were corrected using PLINK’s-flip function. The target dataset was phased using the EPIGEN-5M dataset as a phasing reference. Genotype imputation was performed using IMPUTE2 v2.3.2 [30]. The IMPUTE2 info score was used as a metric of imputation quality, and only variants with an info score ≥ 0.8 and MAF ≥ 1% were retained.

### 2.5. Population Genetic Structure and Linkage Disequilibrium

Individual ancestry patterns were analyzed using ADMIXTURE (version 1.3.0) under unsupervised mode. The analysis incorporated reference populations of European (EUR), African (AFR), and Native American (NAT) ancestries from the 1000 Genomes Project (phase 3). A value of K = 3 was selected, reflecting the primary continental ancestral groups—European, African, and Native American—that contributed to the formation of the Brazilian population [31].

Principal component analysis (PCA) was conducted using the complete dataset of unrelated individuals from the 1000 Genomes Project (phase 3) as a reference. This dataset includes individuals of EUR, AFR, East Asian (EAS), South Asian (SAS), and NAT ancestries. The 1000 Genomes panel was merged with genetic data from the SCAALA cohort, restricting the analysis to autosomal variants with MAF > 0.1 that were shared between both datasets. Data were subsequently pruned using the PLINK software with a window size of 50 markers, a step size of 5, and a variance inflation factor (VIF) threshold of 1.5, resulting in 208,633 markers for PCA calculation. Linkage Disequilibrium (LD, r^2^) analyses were performed using the HAPLOVIEW software (v4.2).

### 2.6. Functional and Pathway Enrichment Analyses

Potentially regulatory SNVs were identified through in silico analysis of the human genome (RefSeq: GRCh38). The positions of SNVs were cross-referenced with DNA sequence annotations (https://www.ensembl.org/index.html, accessed on 15 July 2024), including intron and exon locations, evidence of promoter/enhancer regions, DNAse I hypersensitivity (open chromatin), and eQTL (expression Quantitative Trait Locus; https://gtexportal.org/home/, accessed on 15 July 2024).

Pathway enrichment analysis was conducted using all markers associated with internalizing symptoms at a significance level of *p* < 0.01. Genes were mapped to rsIDs using the g:SNPense tool (https://biit.cs.ut.ee/gprofiler/, accessed on 20 October 2024), resulting in the identification of 2122 gene IDs. These genes were analyzed for pathway overrepresentation using the Kyoto Encyclopedia of Genes and Genomes (KEGG) database. Overrepresentation analyses of genes in canonical pathways were performed with the WebGestalt tool (www.webgestalt.org/, accessed on 20 October 2024). To control for false positives, the False Discovery Rate (FDR) method by Benjamini-Hochberg was applied with a significance threshold set at pFDR < 0.05.

### 2.7. Statistical Analysis

The associations between internalizing symptoms and quantitative or qualitative variables were analyzed using the Mann–Whitney test or Chi-Squared (*χ*^2^) test, respectively. Genome-wide association analysis (GWAS) was performed using multivariate logistic regression under an additive model, adjusting for sex and the first seven principal components (accounting for over 80% of the observed genomic variance). Statistical significance was defined as *p* < 5 × 10^−8^, based on the Bonferroni correction for all common and independent SNVs in the human genome. Variants with 5 × 10^−8^ < *p* < 10^−5^ were considered suggestively associated with internalizing symptoms. The results are presented as estimates of odds ratios (OR) and confidence intervals (CI). Additionally, PLINK’s -clump function was used to identify markers with the lowest *p*-value at each locus, capturing significant or suggestive association signals. These SNVs were grouped considering a maximum physical distance of 250 kb and a linkage disequilibrium threshold (r^2^) of 0.5.

## 3. Results

### 3.1. Characterization of the Study Population

Internalizing symptoms in the Brazilian children were evaluated using the CBCL questionnaire, adapted to Portuguese. Participants were categorized based on their CBCL T-scores, with those scoring < 64 as not exhibiting internalizing symptoms (N-INT), and those scoring ≥ 64 classified as exhibiting internalizing symptoms (INT). Among the 1244 children in the study, 794 were assigned to the N-INT group and 450 to the INT group (Table 1). The proportion of females was significantly higher (*p* < 0.05) in the N-INT group (49.1%) compared to the INT group (39.6%). However, the median ages of the two groups did not differ significantly.

**Figure 1:**
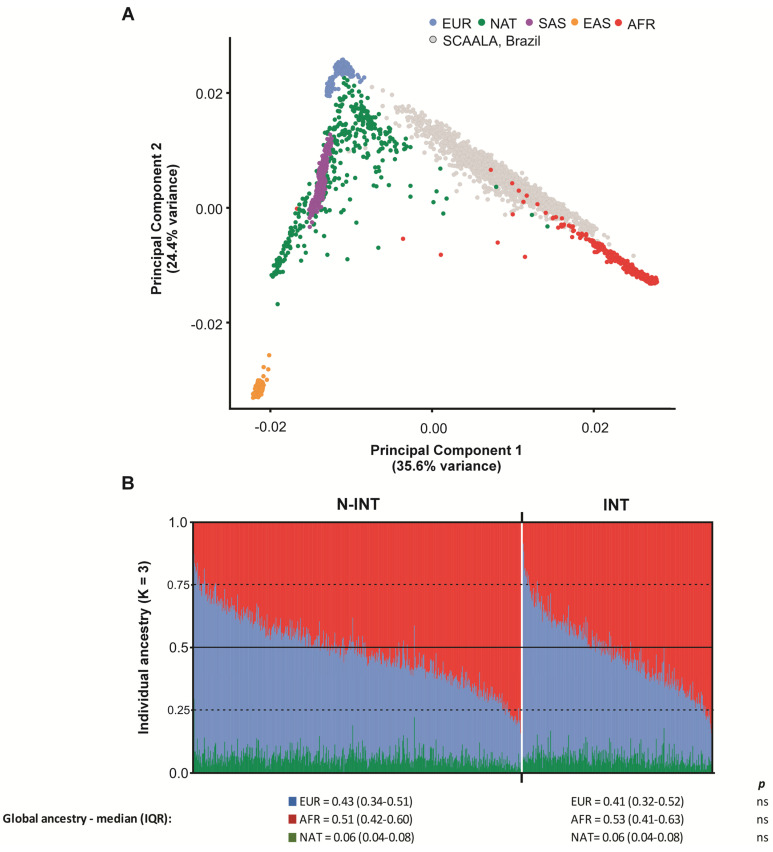
Ancestry analyses of children from the SCAALA cohort. (**A**) Principal Component Analysis (PCA) comparing children from the SCAALA cohort with reference populations from the 1000 Genomes Project. (**B**) Bar plots showing the individual ancestries of the participants (N-INT, n = 794 individuals; INT, n = 450), as determined by the ADMIXTURE method. Abbreviations: N-INT, CBCL T score < 64. INT, CBCL T score ≥ 64; Europeans (EUR), Native Americans (NAT), South Asians (SAS), East Asians (EAS), and Africans (AFR); IQR, interquartile range (first–third quartiles); *p*, the *p*-value for the Mann–Whitney test; ns, not significant.

Principal component analysis shows the admixture patterns of the studied population (Figure 1A). The global ancestry composition of children in the SCAALA cohort is shown in Figure 1B. In the N-INT group, the global ancestry averages are 0.43 (IQR: 0.34–0.51) European, 0.51 (IQR: 0.42–0.60) African, and 0.06 (IQR: 0.04–0.08) Native American. Similarly, children in the INT group exhibit average ancestries of 0.41 (IQR: 0.32–0.52) European, 0.53 (IQR: 0.41–0.63) African, and 0.06 (IQR: 0.04–0.08) Native American.

### 3.2. Genome-Wide Association Analysis

**Figure 2:**
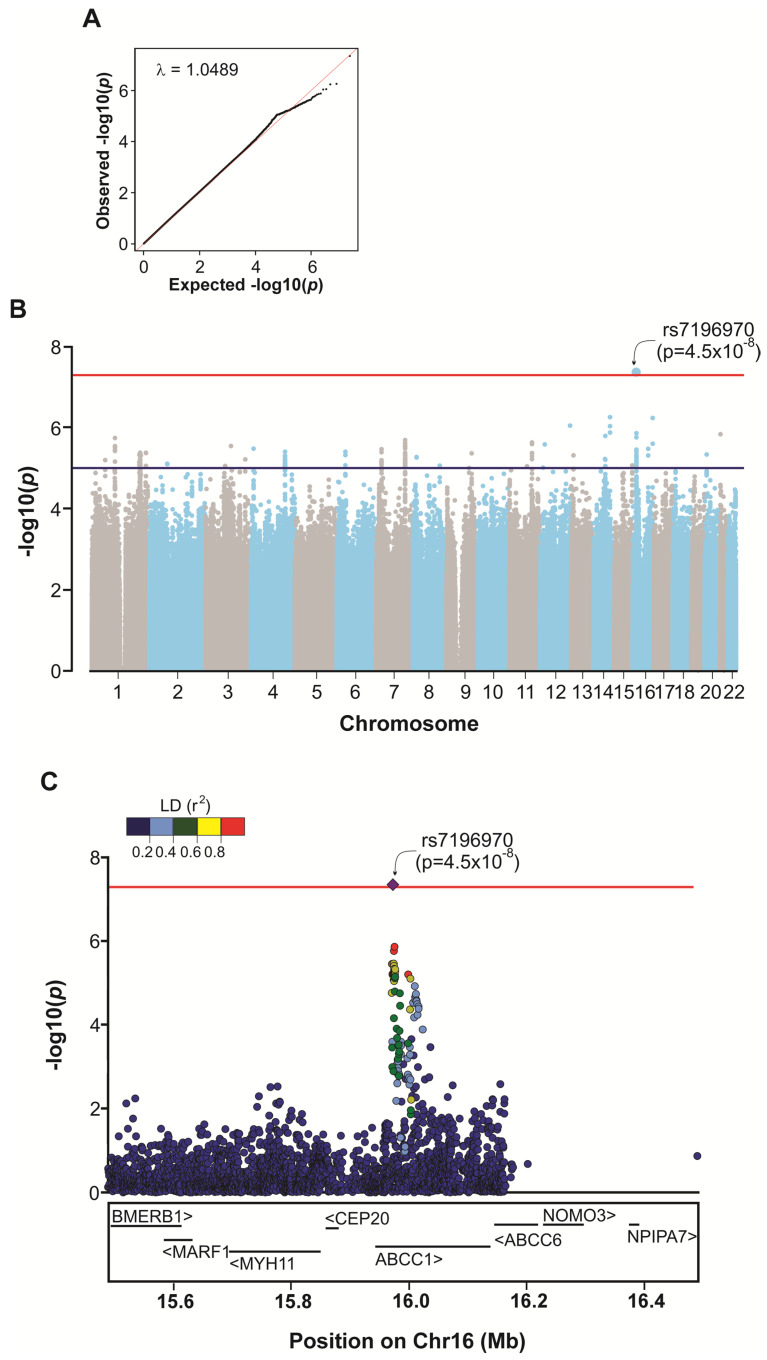
Genome-wide association analysis of internalizing symptoms in children from the SCAALA cohort. (**A**) Quantile–Quantile (QQ) plot showing observed and expected *p*-values. (**B**) Manhattan plot of association statistics obtained from multivariate logistic regression (additive model), with sex and seven principal components included as covariates. The red line represents the genomic significance threshold (*p* < 5 × 10^−8^), while the blue line indicates the threshold for suggestive associations (5 × 10^−8^ < *p* < 10^−5^). (**C**) Regional association plot at the *ABCC1* locus. The plot shows linkage disequilibrium (LD, r^2^) between the lead variant rs7196970 (purple diamond) and other variants (circles) within the region 16:15503151–16239180 (RefSeq: GRCh38). The positions of coding genes within this region are shown at the bottom of the figure.

The association of polymorphisms with internalizing symptoms was investigated. As shown in the Quantile–Quantile plot (Figure 2A), there is no early deviation of observed values from expected values. The estimated genomic inflation factor (λ) was also 1.0489, suggesting that the population’s genomic structure did not significantly affect the association results. This genome-wide association approach revealed that the variant rs7196970 (G), located in an intronic region of the ATP Binding Cassette Subfamily C Member 1 (*ABCC1*) gene (16p13.11), is significantly associated [*p* = 4.5 × 10^−8^, odds ratio (OR) = 0.61, confidence interval (CI) = 0.51–0.73] with internalizing symptoms in the SCAALA cohort (Figure 2B). A closer examination of the region (16:15503151–16239180) reveals that SNVs highly correlated with rs7196970 (r^2^ ≥ 0.8) are located within non-coding sequences of the *ABCC1* gene (Figure 2C).

In total, 220 variants were suggestively associated (5 × 10^−8^ < *p* < 10^−5^) with internalizing symptoms. These SNVs are located in 16 autosomal chromosomes, including regions near the *ABCC1*, *TTC7B*, *GPR88*, *TMEM132C*, *SAMSN1*, *POT1-AS1*, *PLEKHA5*, *NAE1*, *CA7*, *GPNMB*, *LINC00536*, *MIR205*, *TMEM245*, *PLCB1*, *RHEX*, *TUSC3*, *ACTN1*, *NLGN1*, *ADAM21*, *DAB1*, *LYSMD4*, *CAPN5*, and *CORO2A* genes. Table 2 lists 37 index markers (linkage disequilibrium-based clumping; see Section 2) significantly or suggestively associated with internalizing symptoms in Brazilian children.

### 3.3. In Silico Functional Analysis

**Figure 3:**
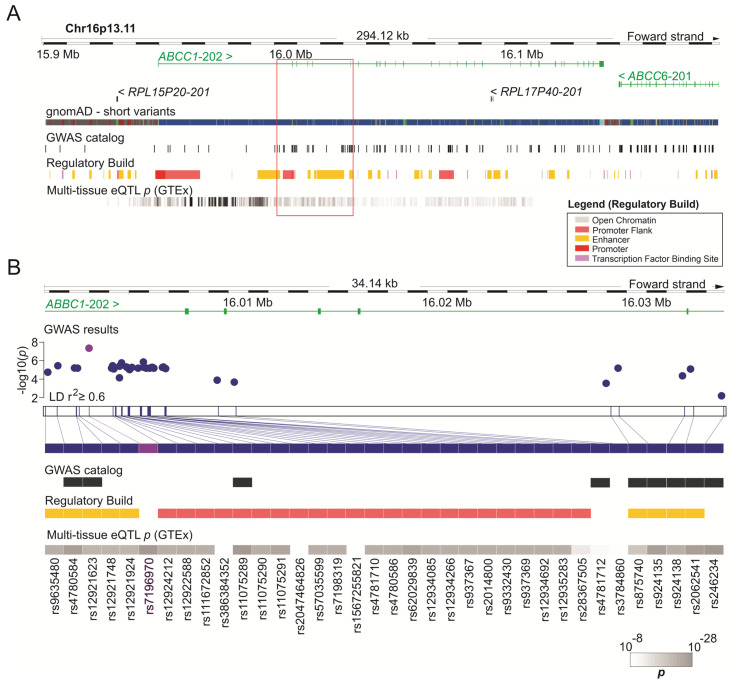
Functional annotation of variants in linkage disequilibrium with rs7196970 (*ABCC1* locus). (**A**) Schematic diagram of the locus containing the *ABCC1* gene. The green solid lines and rectangles represent introns and exons, respectively. This region was cross-referenced with DNA sequence annotations, including short variants from the gnomAD consortium, previous associations with human traits (as recorded in the GWAS catalog platform), regulatory elements, and expression Quantitative Trait Locus (eQTL) data from the GTEx multi-tissue meta-analysis (*p*-value). (**B**) Magnified view of a region within the *ABCC1* gene, highlighting SNVs in moderate to high linkage disequilibrium (r^2^ > 0.6) with rs7196970. Image generated using the Ensembl Genome Browser (http://www.ensembl.org, accessed on 15 July 2024).

Functional annotations were performed for the set of SNVs in moderate to high LD (r^2^ ≥ 0.6) with rs7196970 (Figure 3A). This analysis revealed the potential functional implications of variants at this locus. As shown in Figure 3B, the majority of SNVs within this LD block are located in regions marked by epigenetic signatures of active regulatory elements, such as promoters (H3K4me3) or enhancers (H3K4me1, H3K27ac). As evidenced by the Genotype-Tissue Expression (GTEx) consortium, nearly all of the evaluated SNVs were significantly associated with differential expression of the *ABCC1* gene across several human tissues (GTEx multi-tissue meta-analysis). Furthermore, according to data from the GWAS Catalog platform, nine of these SNVs have been previously identified as significantly associated with other human traits.

### 3.4. Pathway Enrichment Analysis

**Figure 4:**
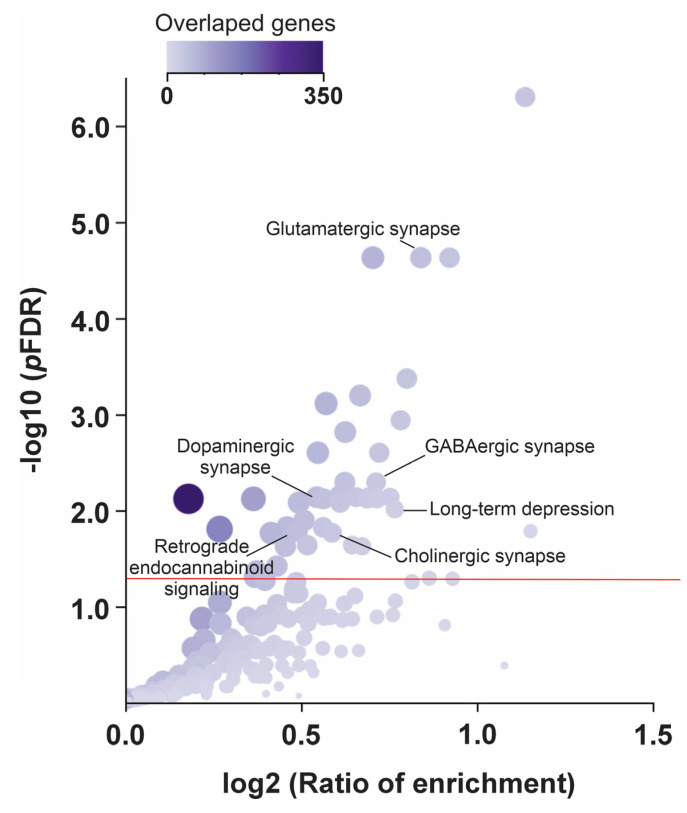
Pathway enrichment analysis for internalizing symptoms in children from the SCAALA cohort. Overrepresentation analysis was conducted on 2122 genes, which were matched with the canonical KEGG pathways. Volcano plot showing the pathways. The red line represents the significance threshold [*p*-value from the false discovery ratio (pFDR) < 0.05]. The size of the dots corresponds to the pathway’s gene set size. The ratio of enrichment is the number of observed genes divided by the number of expected genes from KEGG (according to the WebGestalt tool, www.webgestalt.org/, accessed on 20 October 2024).

A genome-wide pathway enrichment analysis was conducted to identify potential mechanisms associated with internalizing symptoms in Brazilian children. In this context, Genes situated nearest to SNVs with a *p*-value < 0.01 in the GWAS were prioritized for further investigation. This *p*-value threshold was selected to assess the broader genetic contribution to the trait, considering the polygenic nature of internalizing disorders. A list of 2122 genes was cross-referenced with canonical pathway data from the KEGG database, resulting in the identification of 42 significantly enriched pathways (pFDR < 0.05) (Figure 4). Notably, several of these pathways are associated with the nervous system, including Glutamatergic synapse (Rank = 4, pFDR = 2.9 × 10^−7^); GABAergic synapse (Rank = 13, pFDR = 2.0 × 10^−4^); Dopaminergic synapse (Rank = 22, pFDR = 5.4 × 10^−4^); Long-term depression (Rank = 27, pFDR = 8.0 × 10^−4^); Cholinergic synapse (Rank = 29, pFDR = 1.3 × 10^−3^); and Retrograde endocannabinoid signaling (Rank = 32, pFDR = 1.6 × 10^−3^).

## 4. Discussion

In the present study, we explored the genetic architecture of internalizing symptoms in admixed Brazilian children. We identified a genome-wide significant association of rs7196970, located in the *ABCC1* gene, with internalizing symptoms. This variant, along with highly correlated SNVs, is located in epigenetically active regulatory regions and is linked to differential *ABCC1* expression across multiple tissues. Pathway enrichment analysis also revealed significant involvement of genes in neurobiological pathways, including glutamatergic, GABAergic, and dopaminergic synapses, all critical to neuronal signaling and the regulation of mood and behavior. These findings provide evidence for *ABCC1* and associated pathways as contributors to internalizing symptoms in children.

The *ABCC1* gene is a member of the ATP-binding cassette (ABC) superfamily, responsible for transporting molecules across cellular membranes [32]. It is highly expressed in various tissues, including the thymus, parathyroid glands, and skeletal muscle [33]. In the nervous system, the ABCC1 protein plays a critical role in the blood–brain barrier, regulating the influx and efflux of substances to prevent the accumulation of toxins [32]. It also clears β-amyloid, a key molecule linked to Alzheimer’s disease [34]. Elevated levels of β-amyloid and neurofibrillary tangles have been observed in the hippocampus of patients with depression, suggesting a shared pathophysiological mechanism between Alzheimer’s disease and depression [35]. The blood–brain barrier is a crucial regulator of immune, blood, and pathogenic entry into the central nervous system (CNS). Disruptions to its normal function can result in CNS disorders, which may be linked to behavioral changes and neurodegeneration [36,37].

Although direct links between ABCC1 and internalizing disorders are not well established, this protein is known to regulate corticosterone levels, which, similar to cortisol, influence stress-related outcomes [38]. Given corticosterone’s role in stress regulation, ABCC1 could potentially interact with the hypothalamic-pituitary-adrenal (HPA) axis, a well-studied pathway implicated in the development of internalizing behavioral outcomes [39,40,41]. Interestingly, genetic variations in the *ABCC1* gene have been associated with differential patient responses to the antidepressant citalopram [42].

A genetic marker is not necessarily the causal variant; it may instead be in linkage disequilibrium with a functional variant. Then, we analyzed all SNVs moderately or strongly correlated (LD) with rs7196970 to rule out the possibility that polymorphisms in nearby genes might account for the observed associations. A closer examination of the region revealed that all SNVs tagged by rs7196970 were located between introns 1 and 6 of the gene. Notably, the variants rs12921623, rs11075289, rs4781712, rs924135, and rs2062541, all in LD with rs7196970, have been linked to carnitine levels in blood and urine [43,44,45]. Carnitine and its derivatives naturally occur in mammals and are essential cofactors in mitochondrial fatty acid oxidation [46]. Carnitine deficiency in the CNS has been associated with oxidative stress and cognitive decline, highlighting its critical role in maintaining neural health [47,48]. Interestingly, reduced carnitine levels in patients with depression suggest its potential as a biological marker for this disorder [49] or, through its modulatory effects on glutamate, as a candidate for alternative therapeutic strategies [50].

Here, we identified multiple alleles across 16 autosomal chromosomes that exhibit suggestive associations with internalizing symptoms in Brazilian children, emphasizing the polygenic nature of these conditions. These findings highlight contributions from both neurological and systemic pathways to their etiology. Among the implicated genes, G protein-coupled receptor 88 (*GPR88*), primarily expressed in the striatum [51], may influence susceptibility to internalizing disorders through its role in motivation and reward processing, pathways often disrupted in depressive states [52]. Structural and functional abnormalities in the striatum are well-documented in depression [53]; altered GPR88 expression has been linked to learning deficits and neuropsychiatric disorders [54,55]. Additionally, the immune-related SAM domain, SH3 domain, and nuclear localization signals 1 (*SAMSN1*) gene, predominantly expressed in immune cells [56], underscores the link between systemic inflammation and neurological dysfunction. Chronic inflammation and disruptions of the blood–brain barrier may facilitate peripheral immune cell infiltration into the central nervous system, potentially exacerbating mental disorders [57]. Finally, neuronal precursor cell-expressed developmentally down-regulated protein 8 activating enzyme 1 (*NAE1*) may contribute to neuronal differentiation and synaptic formation via its involvement in the neddylation pathway, which is essential for synaptic plasticity and has been implicated in neurodegenerative processes [58,59]. These diverse pathways collectively illustrate the complex interplay between genetic, neurological, and systemic factors potentially underlying internalizing symptoms.

Our genome-wide pathway enrichment analysis underscores the potential role of several key neural pathways in childhood internalizing disorders. While glutamatergic signaling has traditionally been implicated in neuronal hyperexcitability [60], dysregulation of this system has been linked to structural and functional changes in the prefrontal cortex and hippocampus, which are brain regions crucial for emotional regulation [61,62]. Similarly, alterations in GABAergic inhibitory signaling can impair the balance between excitatory and inhibitory neurotransmission, contributing to heightened anxiety and vulnerability to stress [63,64]. The cholinergic system, known for its role in cognitive processes, has also been associated with neural plasticity and stress responses, making it a candidate pathway for internalizing symptoms [65,66]. The dopaminergic pathway, while classically linked to reward processing, is also recognized for its role in mood regulation and anhedonia, which are core features of internalizing disorders [67]. Additionally, the enrichment of the retrograde endocannabinoid signaling pathway in our analysis aligns with evidence that this system modulates synaptic plasticity and emotional responses, particularly in stress-related contexts [68]. Finally, the long-term depression pathway may also contribute to the dysregulation of neural circuits implicated in the persistence and exacerbation of internalizing disorders [69]. These findings collectively highlight the complex interactions between neurotransmitter systems and synaptic mechanisms in the etiology of childhood internalizing disorders. They offer valuable insights for future research and therapeutic strategies, particularly in developing more targeted treatments. Focusing on these pathways may help address underlying neurobiological dysregulations, enabling personalized interventions that target the fundamental mechanisms of childhood internalizing disorders.

Although this study provides valuable insights into the genetic mechanisms underlying childhood internalizing symptoms, some limitations must be considered. First, the relatively small sample size may have limited the statistical power to detect association signals from variants with small effect sizes. Second, the findings are based on a single cohort from Salvador, Brazil, which may restrict their generalizability to other populations. Replication efforts are particularly challenging due to the limited number of genome-wide studies on childhood internalizing disorders, especially due to the lack of studies focused on non-European populations. Finally, while the genetic associations identified are promising, functional validation of these loci is critical to fully understand their biological significance.

Considering the multifactorial nature of internalizing disorders, future studies should focus on replicating these findings in larger, well-characterized, and more diverse cohorts, particularly across other Latin American populations. Finally, whole-genome sequencing, which accounts for common and rare variants, combined with multi-omic data, will provide a more comprehensive view of the molecular pathways involved in childhood internalizing disorders. Integrating these data into public health strategies may be crucial for early screening and prevention programs, while also reducing the long-term impact of these disorders across the lifespan.

## 5. Conclusions

Our study provides insights into the genetic mechanisms of childhood internalizing disorders in an admixed Latin American population, with a particular focus on variants in the *ABCC1* gene and associated neurobiological pathways. These findings could have implications for understanding the progression of these disorders into adulthood and for developing targeted interventions.

## Figures and Tables

**Table 1:** Demographic characteristics of the studied sample.

	N-INT	INT	*p*
Number of children (%)	794 (63.8)	450 (36.2)	-
Sex, female (%)	390 (49.1)	178 (39.6)	<0.05
Median age, years (IQR)	8 (6–9)	8 (7–9)	ns

Abbreviations: N-INT, no internalizing symptoms (CBCL T score < 64); INT, internalizing symptoms (CBCL T score ≥ 64); IQR, interquartile range (first–third quartiles); *p*, *p*-value for association tests (Mann–Whitney or χ^2^ for quantitative or qualitative variables, respectively); ns, not significant.

**Table 2:** List of the top SNVs at loci showing significant or suggestive association signals with internalizing symptoms in children from Salvador, Brazil.

SNP	Coordinate ^a^	Locus	A1	MAF	OR	95% CI	*p*
rs7196970	16:16002935	16p13.11	G	0.49	0.61	0.51–0.73	4.5 × 10^−8^
rs11847624	14:90740595	14q32.11	G	0.21	1.66	1.36–2.02	5.5 × 10^−7^
rs1728386	16:86384206	16q24.1	T	0.24	1.65	1.35–2.00	5.8 × 10^−7^
rs138365190	12:128452629	12q24.32	A	0.03	3.13	1.98–4.93	8.9 × 10^−7^
rs405792	21:14494814	21q11.2	A	0.25	0.61	0.50–0.75	1.5 × 10^−6^
rs11166475	1:100556556	1p21.2	A	0.41	1.52	1.28–1.81	1.8 × 10^−6^
rs1526415	7:125439821	7q31.33	A	0.06	2.33	1.64–3.30	2.0 × 10^−6^
rs33973779	11:98784980	11q22.1	G	0.09	1.96	1.48–2.59	2.3 × 10^−6^
rs57145395	7:125377403	7q31.33	G	0.06	2.19	1.58–3.03	2.4 × 10^−6^
rs74894866	12:19026261	12p12.3	T	0.20	0.58	0.47–0.73	2.6 × 10^−6^
rs79063512	4:11194663	4p16.1	T	0.07	2.11	1.54–2.89	3.3 × 10^−6^
rs152168	16:66852656	16q22.1	A	0.30	0.64	0.53–0.77	3.3 × 10^−6^
rs156426	7:23267856	7p15.3	C	0.07	2.13	1.55–2.94	3.4 × 10^−6^
rs2082027	4:147412952	4q31.22	C	0.34	0.66	0.55–0.78	3.9 × 10^−6^
rs115631938	6:37537257	6p21.2	C	0.04	2.69	1.77–4.08	3.9 × 10^−6^
rs2179654	1:209434426	1p32.2	T	0.25	1.57	1.29–1.90	4.2 × 10^−6^
rs115162927	1:234572600	1q42.2	A	0.02	4.16	2.27–7.64	4.2 × 10^−6^
rs57279798	9:109082533	9q31.3	A	0.08	2.00	1.49–2.68	4.3 × 10^−6^
rs73092035	20:8440666	20p12.3	C	0.18	0.57	0.45–0.73	4.6 × 10^−6^
1:206062625_T	1:206062625	1q32.1	C	0.41	1.48	1.25–1.76	4.7 × 10^−6^
rs1924622	13:28522605	13q12.3	C	0.07	2.04	1.51–2.78	4.8 × 10^−6^
rs76680358	1:212556035	1q32.3	T	0.16	1.69	1.35–2.12	4.9 × 10^−6^
rs113284492	7:125396505	7q31.33	T	0.02	3.79	2.14–6.71	4.9 × 10^−6^
rs6665232	1:209512951	1p32.2	A	0.48	0.68	0.57–0.80	5.1 × 10^−6^
rs12682188	8:15772149	8p22	C	0.39	0.66	0.56–0.79	5.3 × 10^−6^
rs10220411	14:68985371	14q24.1	G	0.36	1.49	1.25–1.77	5.9 × 10^−6^
rs626337	3:173442741	3q26.31	A	0.47	0.68	0.58–0.80	6.1 × 10^−6^
rs1959485	14:70487504	14q24.2	T	0.40	0.67	0.57–0.80	6.2 × 10^−6^
rs515683	1:58183297	1p32.2	A	0.37	0.67	0.56–0.80	6.4 × 10^−6^
rs78294387	7:23289364	7p15.3	A	0.10	1.85	1.41–2.41	7.3 × 10^−6^
2:78015566_C	2:78015566	2p12	C	0.27	1.54	1.27–1.86	7.8 × 10^−6^
rs73466526	15:99736749	15q26.3	A	0.12	1.73	1.36–2.21	8.6 × 10^−6^
rs7011010	8:116114085	8q23.3	C	0.36	1.48	1.25–1.76	8.7 × 10^−6^
rs1983270	3:86304891	3p12.1	T	0.23	1.58	1.29–1.93	8.8 × 10^−6^
rs4945142	11:77095476	11q13.5	T	0.35	1.48	1.25–1.76	9.0 × 10^−6^
rs11054328	12:11510821	12p13.2	T	0.15	1.69	1.34–2.13	9.7 × 10^−6^
rs701379	9:98124543	9q22.33	T	0.26	0.64	0.53–0.78	9.9 × 10^−6^

Abbreviations: SNV, Single Nucleotide Variant; A1, reference allele; MAF, Minor Allele Frequency; OR, odds ratio; 95% CI, 95% confidence interval; *p*, *p*-value (association test). ^a^ Coordinate, Chromosome:base pair (RefSeq: GRCh38).

## Data Availability

The EPIGEN data are deposited in the European Nucleotide Archive [PRJEB9080 (ERP010139) Genomic Epidemiology of Complex Diseases in Population-Based Brazilian Cohorts], with accession number EGAS00001001245.
